# Supplementary material for: Computational and In Vitro Investigation of (-)-Epicatechin and Proanthocyanidin B2 as Inhibitors of Human Matrix Metalloproteinase 1
Source: Biomolecules. 2020 Sep 28;10(10):1379. doi: 10.3390/biom10101379 (PMC7650666; doi:10.3390/biom10101379)
Supplement: Supplementary file 1 [file biomolecules-10-01379-s001.pdf]

Computational and In Vitro Investigation of (-)-Epicatechin and Proanthocyanidin B2 as Inhibitors of Human Matrix Metalloproteinase 1

Kyung Eun Lee [1,†], Shiv Bharadwaj [1,†], Umesh Yadava [2] and Sang Gu Kang [1,3,*]

1. Department of Biotechnology, Institute of Biotechnology, College of Life and Applied Sciences, Yeungnam University, 280 Daehak-Ro, Gyeongsan, Gyeongbuk 38541, Korea; keun126@ynu.ac.kr (K.E.L.); shiv@ynu.ac.kr (S.B.)
2. Department of Physics, Deen Dayal Upadhyay Gorakhpur University, Gorakhpur, Uttar Pradesh 273009, India; u_yadava@yahoo.com
3. Stemforce, 313 Institute of Industrial Technology, Yeungnam University, 280 Daehak-Ro, Gyeongsan, Gyeongbuk 38541, Korea

* Correspondence: kangsg@ynu.ac.kr

† These authors contributed equally to this work.

S1. Results and Discussion

S1.1. ADME and Quantum chemical calculation analysis

Table S1. ADME analysis for the bioactive compounds against MMP-1.

Molecule	Epicatechin	Procyanidin B2	Epigallocatechin Gallate
Formula	$C_{15}H_{14}O_6$	$C_{30}H_{26}O_{12}$	$C_{22}H_{18}O_{11}$
MW	290.27	578.52	458.37
#Heavy atoms	21	42	33
#Aromatic heavy atoms	12	24	18
Fraction Csp3	0.2	0.2	0.14
#Rotatable bonds	1	3	4
#H-bond acceptors	6	12	11
#H-bond donors	5	10	8
MR	74.33	146.71	112.06
TPSA	110.38	220.76	197.37
iLOGP	1.47	1.35	1.53
XLOGP3	0.36	2.37	1.17
WLOGP	1.22	2.35	1.91
MLOGP	0.24	-0.26	-0.44
Silicos-IT Log P	0.98	1.14	0.57
Consensus Log P	0.85	1.39	0.95
ESOL Log S	-2.22	-5.14	-3.56
ESOL Solubility (mg/ml)	1.74E+00	4.15E-03	1.27E-01
ESOL Solubility (mol/l)	5.98E-03	7.17E-06	2.76E-04
ESOL Class	Soluble	Moderately soluble	Soluble
Ali Log S	-2.24	-6.65	-4.91
Ali Solubility (mg/ml)	1.66E+00	1.31E-04	5.64E-03
Ali Solubility (mol/l)	5.72E-03	2.26E-07	1.23E-05
Ali Class	Soluble	Poorly soluble	Moderately soluble
Silicos-IT LogSw	-2.14	-3.91	-2.5
Silicos-IT Solubility (mg/ml)	2.09E+00	7.05E-02	1.46E+00
Silicos-IT Solubility (mol/l)	7.19E-03	1.22E-04	3.18E-03
Silicos-IT class	Soluble	Soluble	Soluble
GI absorption	High	Low	Low
BBB permeant	No	No	No

Pgp substrate	Yes	No	No
CYP1A2 inhibitor	No	No	No
CYP2C19 inhibitor	No	No	No
CYP2C9 inhibitor	No	No	No
CYP2D6 inhibitor	No	No	No
CYP3A4 inhibitor	No	Yes	No
log Kp (cm/s)	-7.82	-8.15	-8.27
Lipinski #violations	0	3	2
Ghose #violations	0	2	0
Veber #violations	0	1	1
Egan #violations	0	1	1
Muegge #violations	0	3	3
Bioavailability Score	0.55	0.17	0.17
PAINS #alerts	1	1	1
Brenk #alerts	1	1	1
Leadlikeness #violations	0	1	1
Synthetic Accessibility	3.5	5.32	4.2

Table S2. Optimized geometry coordinates for the compound (-)-epicatechin.

S.no.	Atom	Bond atom	Bond length (Å)	Angle atom	Angle (º)	2nd angle Atom	2nd angle (º)	2nd angle type
1	C(10)	-	-	-	-	-	-	-
2	C(11)	C(10)	1.398			-	-	-
3	O(1)	C(11)	1.37	C(10)	122.417	-	-	-
4	C(14)	C(11)	1.4	C(10)	122.176	O(1)	115.404	Pro-S
5	C(9)	C(10)	1.509	C(11)	121.369	O(1)	-1.666	Dihedral
6	C(13)	C(10)	1.404	C(11)	117.041	C(9)	121.569	Pro-R
7	C(17)	C(13)	1.394	C(10)	122.238	C(11)	0.431	Dihedral
8	C(18)	C(14)	1.392	C(11)	118.962	C(10)	0.261	Dihedral
9	C(7)	C(9)	1.527	C(10)	111.155	C(11)	-15.131	Dihedral
10	C(8)	O(1)	1.432	C(11)	117.769	C(10)	-13.993	Dihedral
11	C(12)	C(8)	1.512	O(1)	107.737	C(7)	112.382	Pro-S
12	C(15)	C(12)	1.403	C(8)	119.631	O(1)	149.961	Dihedral
13	C(16)	C(12)	1.396	C(15)	118.776	C(8)	121.488	Pro-R
14	C(19)	C(15)	1.392	C(12)	121.475	C(16)	0.018	Dihedral
15	C(20)	C(16)	1.399	C(12)	120.046	C(15)	-0.227	Dihedral
16	C(21)	C(19)	1.411	C(15)	119.459	C(12)	0.026	Dihedral
17	O(3)	C(13)	1.37	C(10)	116.083	C(17)	121.679	Pro-S
18	O(4)	C(18)	1.368	C(14)	122.395	C(17)	116.922	Pro-S
19	O(5)	C(19)	1.364	C(15)	123.55	C(21)	116.99	Pro-S
20	O(6)	C(21)	1.364	C(19)	117.069	C(20)	123.854	Pro-S
21	H(26)	C(14)	1.086	C(11)	119.095	C(18)	121.942	Pro-S
22	H(27)	C(15)	1.089	C(12)	119.756	C(19)	118.767	Pro-S
23	H(29)	C(16)	1.084	C(12)	119.601	C(20)	120.317	Pro-R
24	H(30)	C(17)	1.086	C(13)	121.629	C(18)	119.473	Pro-S
25	H(31)	C(20)	1.088	C(16)	119.809	C(21)	119.023	Pro-R
26	O(2)	C(7)	1.417	C(8)	111.549	C(9)	107.779	Pro-S
27	H(22)	C(7)	1.102	O(2)	110.97	C(8)	107.349	Pro-R
28	H(23)	C(8)	1.101	O(1)	108.844	C(7)	107.425	Pro-R
29	H(24)	C(9)	1.098	C(7)	109.251	C(10)	110.837	Pro-R
30	H(25)	C(9)	1.094	C(7)	108.699	C(10)	110.399	Pro-S
31	H(28)	O(2)	0.969	C(7)	107.633	C(8)	51.604	Dihedral
32	H(32)	O(3)	0.966	C(13)	108.832	C(10)	-177.182	Dihedral
33	H(33)	O(4)	0.966	C(18)	108.732	C(14)	0.897	Dihedral
34	H(34)	O(5)	0.966	C(19)	108.79	C(15)	-0.144	Dihedral
35	H(35)	O(6)	0.966	C(21)	108.658	C(19)	-179.225	Dihedral

Table S3. Optimized geometry coordinates for the compound proanthocyanidin B2.

S.no.	Atom	Bond atom	Bond length (Å)	Angle atom	Angle (º)	2nd angle Atom	2nd angle (º)	2nd angle type
1	C(17)	-	-	-	-	-	-	-
2	C(23)	C(17)	1.409	-	-	-	-	-
3	O(1)	C(23)	1.367	C(17)	122.734	-	-	-
4	C(30)	C(23)	1.397	C(17)	122.339	O(1)	114.906	Pro-S
5	C(13)	C(17)	1.522	C(23)	120.848	O(1)	1.116	Dihedral
6	C(26)	C(17)	1.4	C(23)	116.192	C(13)	122.932	Pro-R

7	C(31)	C(26)	1.395	C(17)	122.91	C(23)	-1.434	Dihedral
8	C(33)	C(30)	1.391	C(23)	119.331	C(17)	-0.155	Dihedral
9	C(15)	C(13)	1.531	C(17)	116.548	C(23)	107.377	Dihedral
10	C(18)	C(15)	1.414	C(13)	118.896	C(17)	143.717	Dihedral
11	C(19)	C(18)	1.398	C(15)	123.992	C(13)	-178.379	Dihedral
12	C(24)	C(15)	1.404	C(18)	115.643	C(13)	125.459	Pro-R
13	O(2)	C(18)	1.368	C(19)	120.134	C(15)	115.865	Pro-S
14	C(22)	C(19)	1.507	C(18)	118.762	O(2)	-6.097	Dihedral
15	C(27)	C(19)	1.396	C(18)	117.711	C(22)	123.398	Pro-R
16	C(28)	C(24)	1.399	C(15)	121.994	C(18)	-1.475	Dihedral
17	C(14)	C(13)	1.552	C(17)	108.465	C(15)	112.87	Pro-R
18	C(16)	O(1)	1.445	C(23)	118.836	C(17)	-12.401	Dihedral
19	C(25)	C(16)	1.51	O(1)	107.934	C(14)	114.088	Pro-S
20	C(32)	C(25)	1.402	C(16)	118.798	O(1)	146.601	Dihedral
21	C(34)	C(25)	1.397	C(32)	118.835	C(16)	122.326	Pro-R
22	C(37)	C(32)	1.389	C(25)	120.48	C(34)	-0.535	Dihedral
23	C(38)	C(34)	1.396	C(25)	120.734	C(32)	-0.164	Dihedral
24	C(39)	C(37)	1.405	C(32)	120.478	C(25)	0.725	Dihedral
25	C(20)	C(22)	1.53	C(19)	108.811	C(18)	-35.146	Dihedral
26	C(21)	O(2)	1.448	C(18)	121.99	C(19)	25.433	Dihedral
27	C(29)	C(21)	1.516	O(2)	107.394	C(20)	112.717	Pro-S
28	C(35)	C(29)	1.401	C(21)	118.459	O(2)	-37.839	Dihedral
29	C(36)	C(29)	1.395	C(35)	118.796	C(21)	122.73	Pro-S
30	C(40)	C(35)	1.391	C(29)	121.904	C(36)	-2.476	Dihedral
31	C(41)	C(36)	1.4	C(29)	119.741	C(35)	0.311	Dihedral
32	C(42)	C(40)	1.41	C(35)	119.109	C(29)	3.144	Dihedral
33	O(5)	C(24)	1.372	C(15)	118.163	C(28)	119.841	Pro-S
34	O(6)	C(27)	1.369	C(19)	117.278	C(28)	121.989	Pro-R
35	O(7)	C(26)	1.39	C(17)	117.646	C(31)	119.429	Pro-S
36	O(8)	C(33)	1.366	C(30)	117.446	C(31)	122.256	Pro-R
37	O(9)	C(37)	1.379	C(32)	124.503	C(39)	115.014	Pro-S
38	O(10)	C(39)	1.364	C(37)	120.492	C(38)	120.407	Pro-S
39	O(11)	C(40)	1.362	C(35)	122.033	C(42)	118.858	Pro-R
40	O(12)	C(42)	1.365	C(40)	117.404	C(41)	123.592	Pro-R
41	H(50)	C(28)	1.089	C(24)	119.805	C(27)	120.297	Pro-S
42	H(52)	C(30)	1.083	C(23)	119.955	C(33)	120.713	Pro-S
43	H(53)	C(31)	1.088	C(26)	120.19	C(33)	120.866	Pro-S
44	H(54)	C(32)	1.089	C(25)	120.062	C(37)	119.458	Pro-S
45	H(55)	C(34)	1.083	C(25)	119.497	C(38)	119.763	Pro-R
46	H(57)	C(35)	1.083	C(29)	119.647	C(40)	118.395	Pro-R
47	H(58)	C(36)	1.086	C(29)	120.682	C(41)	119.571	Pro-S
48	H(62)	C(38)	1.085	C(34)	121.182	C(39)	118.451	Pro-S
49	H(63)	C(41)	1.088	C(36)	119.746	C(42)	118.856	Pro-S
50	O(3)	C(14)	1.418	C(13)	111.441	C(16)	111.558	Pro-R
51	O(4)	C(20)	1.417	C(21)	113.064	C(22)	106.972	Pro-S
52	H(43)	C(13)	1.091	C(14)	104.107	C(15)	105.796	Pro-R
53	H(44)	C(14)	1.093	O(3)	105.831	C(13)	109.146	Pro-S
54	H(45)	C(16)	1.095	O(1)	107.978	C(14)	108.172	Pro-R
55	H(46)	C(20)	1.102	O(4)	110.707	C(21)	106.33	Pro-R
56	H(47)	C(21)	1.097	O(2)	106.142	C(20)	107.298	Pro-R
57	H(48)	C(22)	1.099	C(19)	111.74	C(20)	109.068	Pro-S
58	H(49)	C(22)	1.092	C(19)	110.369	C(20)	109.515	Pro-R
59	H(51)	O(3)	0.967	C(14)	107.648	C(13)	63.421	Dihedral
60	H(56)	O(4)	0.968	C(20)	107.538	C(21)	48.36	Dihedral
61	H(59)	O(5)	0.966	C(24)	109.156	C(15)	176.992	Dihedral
62	H(60)	O(6)	0.966	C(27)	109.073	C(19)	-175.456	Dihedral
63	H(61)	O(7)	0.967	C(26)	108.551	C(17)	150.727	Dihedral
64	H(64)	O(8)	0.966	C(33)	109.207	C(30)	179.009	Dihedral
65	H(65)	O(9)	0.965	C(37)	109.685	C(32)	4.03	Dihedral
66	H(66)	O(10)	0.969	C(39)	107.341	C(37)	0.981	Dihedral
67	H(67)	O(11)	0.973	C(40)	107.431	C(35)	11.531	Dihedral
68	H(68)	O(12)	0.966	C(42)	108.663	C(40)	177.995	Dihedral

Table S4. Optimized geometry coordinates for the compound EGCG.

S.no.	Atom	Bond atom	Bond length (Å)	Angle atom	Angle (º)	2nd angle Atom	2nd angle (º)	2nd angle type
1	C(15)	-	-	-	-	-	-	-
2	C(17)	C(15)	1.399	-	-	-	-	-
3	O(1)	C(17)	1.369	C(15)	122.375	-	-	-
4	C(19)	C(17)	1.401	C(15)	122.061	O(1)	115.556	Pro-S
5	C(14)	C(15)	1.51	C(17)	121.33	O(1)	-1.255	Dihedral
6	C(18)	C(15)	1.405	C(17)	117.145	C(14)	121.506	Pro-R
7	C(22)	C(18)	1.393	C(15)	122.178	C(17)	0.535	Dihedral
8	C(23)	C(19)	1.393	C(17)	118.979	C(15)	0.461	Dihedral
9	C(12)	C(14)	1.528	C(15)	110.96	C(17)	-14.637	Dihedral

10	C(13)	O(1)	1.437	C(17)	117.525	C(15)	-15.324	Dihedral
11	O(2)	C(12)	1.443	C(13)	108.011	C(14)	108.886	Pro-S
12	C(16)	C(13)	1.51	O(1)	108.086	C(12)	114.428	Pro-S
13	C(20)	C(16)	1.398	C(13)	119.545	O(1)	139.75	Dihedral
14	C(21)	C(16)	1.398	C(20)	119.47	C(13)	120.956	Pro-R
15	C(25)	C(21)	1.392	C(16)	119.331	C(20)	0.336	Dihedral
16	C(26)	C(20)	1.398	C(16)	121.01	C(21)	-0.612	Dihedral
17	C(27)	C(25)	1.401	C(21)	121.744	C(16)	0.112	Dihedral
18	C(24)	O(2)	1.36	C(12)	118.004	C(13)	123.657	Dihedral
19	C(28)	C(24)	1.484	O(2)	111.753	C(12)	-178.85	Dihedral
20	C(29)	C(28)	1.4	C(24)	117.61	O(2)	176.099	Dihedral
21	C(30)	C(28)	1.402	C(29)	120.165	C(24)	122.224	Pro-S
22	C(31)	C(30)	1.394	C(28)	120.366	C(29)	0.101	Dihedral
23	C(32)	C(29)	1.388	C(28)	119.166	C(30)	0.252	Dihedral
24	C(33)	C(31)	1.405	C(30)	119.814	C(28)	-0.435	Dihedral
25	O(3)	C(18)	1.369	C(15)	115.994	C(22)	121.827	Pro-S
26	O(4)	C(23)	1.367	C(19)	122.426	C(22)	116.848	Pro-S
27	O(5)	C(25)	1.377	C(21)	123.969	C(27)	114.287	Pro-R
28	O(6)	C(26)	1.365	C(20)	123.302	C(27)	116.936	Pro-S
29	O(8)	C(27)	1.362	C(25)	121.313	C(26)	120.005	Pro-R
30	O(9)	C(31)	1.365	C(30)	123.448	C(33)	116.738	Pro-R
31	O(10)	C(32)	1.376	C(29)	124.528	C(33)	114.098	Pro-R
32	O(11)	C(33)	1.356	C(31)	119.769	C(32)	121.118	Pro-S
33	H(38)	C(19)	1.086	C(17)	119.047	C(23)	121.969	Pro-S
34	H(39)	C(20)	1.088	C(16)	120.045	C(26)	118.945	Pro-S
35	H(40)	C(21)	1.085	C(16)	120.09	C(25)	120.576	Pro-R
36	H(41)	C(22)	1.086	C(18)	121.652	C(23)	119.439	Pro-R
37	H(44)	C(29)	1.085	C(28)	119.143	C(32)	121.69	Pro-S
38	H(45)	C(30)	1.085	C(28)	119.588	C(31)	120.041	Pro-S
39	O(7)	C(24)	1.216	O(2)	123.854	C(28)	124.393	Pro-R
40	H(34)	C(12)	1.091	O(2)	108.506	C(13)	109.949	Pro-R
41	H(35)	C(13)	1.101	O(1)	108.485	C(12)	106.312	Pro-R
42	H(36)	C(14)	1.099	C(12)	108.729	C(15)	110.943	Pro-R
43	H(37)	C(14)	1.094	C(12)	109.447	C(15)	110.628	Pro-S
44	H(42)	O(3)	0.966	C(18)	108.991	C(15)	-178.075	Dihedral
45	H(43)	O(4)	0.966	C(23)	108.873	C(19)	-0.697	Dihedral
46	H(46)	O(5)	0.965	C(25)	109.557	C(21)	-7.146	Dihedral
47	H(47)	O(6)	0.966	C(26)	108.639	C(20)	1.625	Dihedral
48	H(48)	O(8)	0.969	C(27)	106.81	C(25)	-1.175	Dihedral
49	H(49)	O(9)	0.966	C(31)	108.571	C(30)	-3.461	Dihedral
50	H(50)	O(10)	0.965	C(32)	109.755	C(29)	0.854	Dihedral
51	H(51)	O(11)	0.97	C(33)	107.15	C(31)	-179.284	Dihedral

S1.2. Molecular docking simulation and intermolecular interaction analysis.

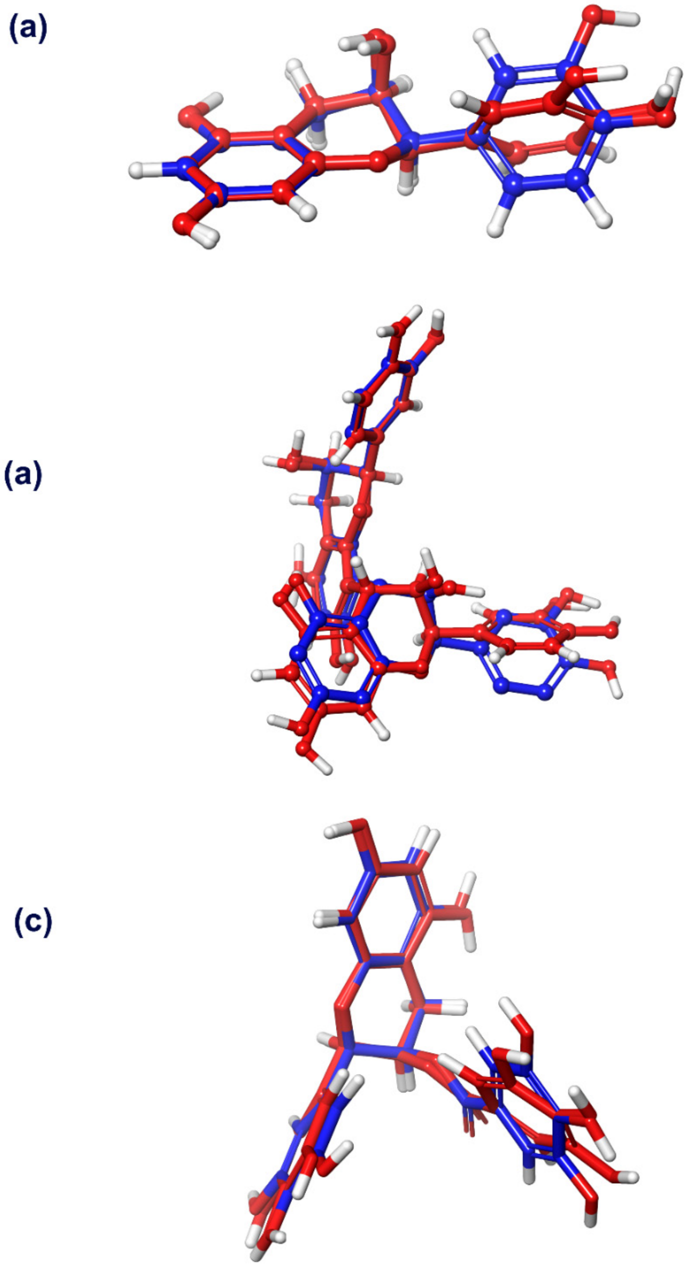

Figure S1. Structural alignment for the B3LYP/6-31G** optimized structural geometries (in red color) with docked conformations (in blue color) of (a) Epicatechin, (b) Proanthocyanidin B2, and reference compound EGCG.

S1.3. Molecular dynamics simulation analysis.

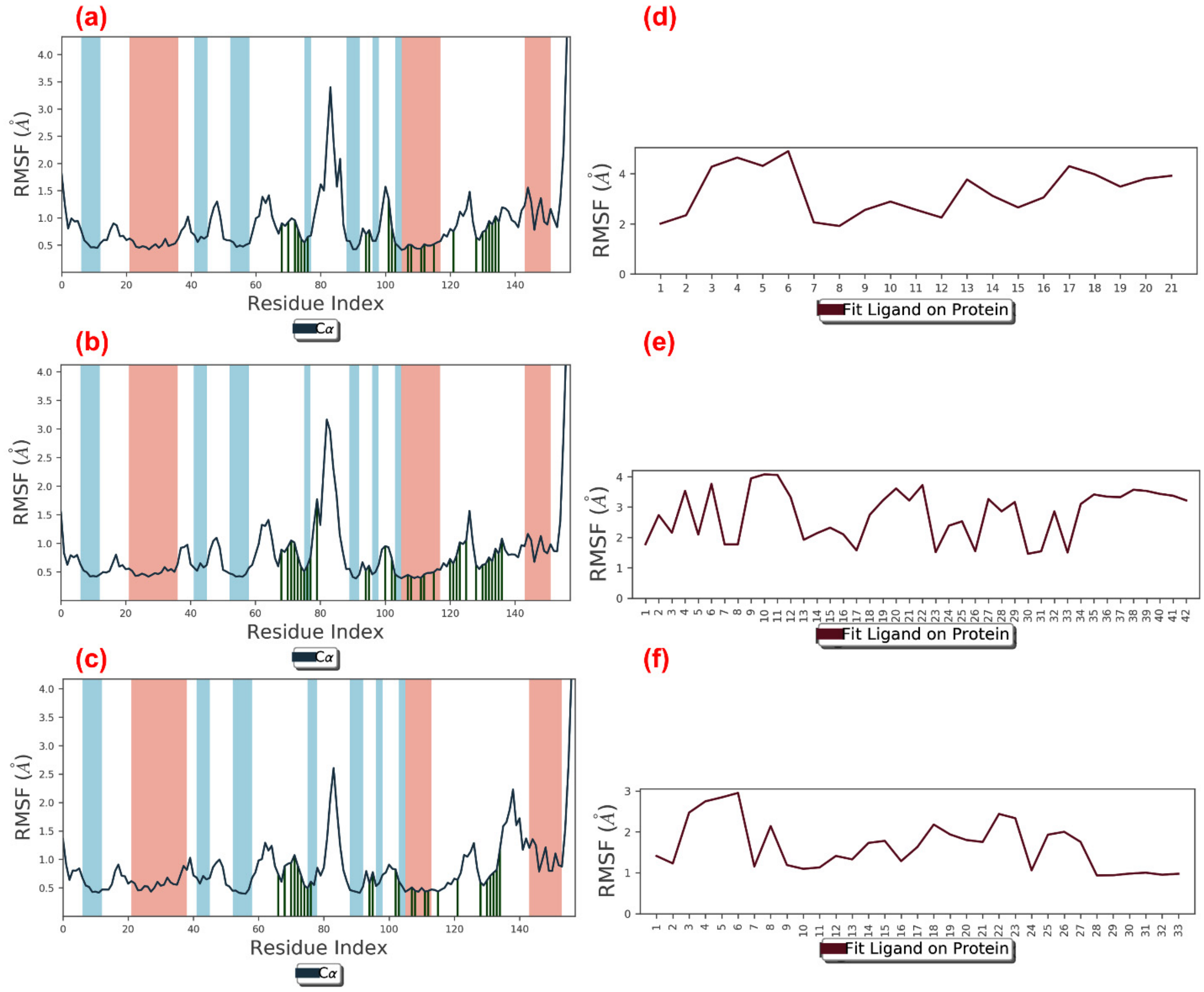

Figure S2. RMSF values calculated for the MMP1 docked with (a) (-)-epicatechin, (b) proanthocyanidin B2, and (c) EGCG during the 500 ns MD simulation. Herein, alpha-helical, and beta-strand regions are highlighted in red and blue backgrounds, respectively with vertical green lines represent the ligand contacts with the residues during the simulation interval. Also, Fit ligand on protein RMSF values were calculated for (d) (-)-epicatechin, (e) proanthocyanidin B2, and (f) EGCG extracted from 500 ns MD simulation trajectories.

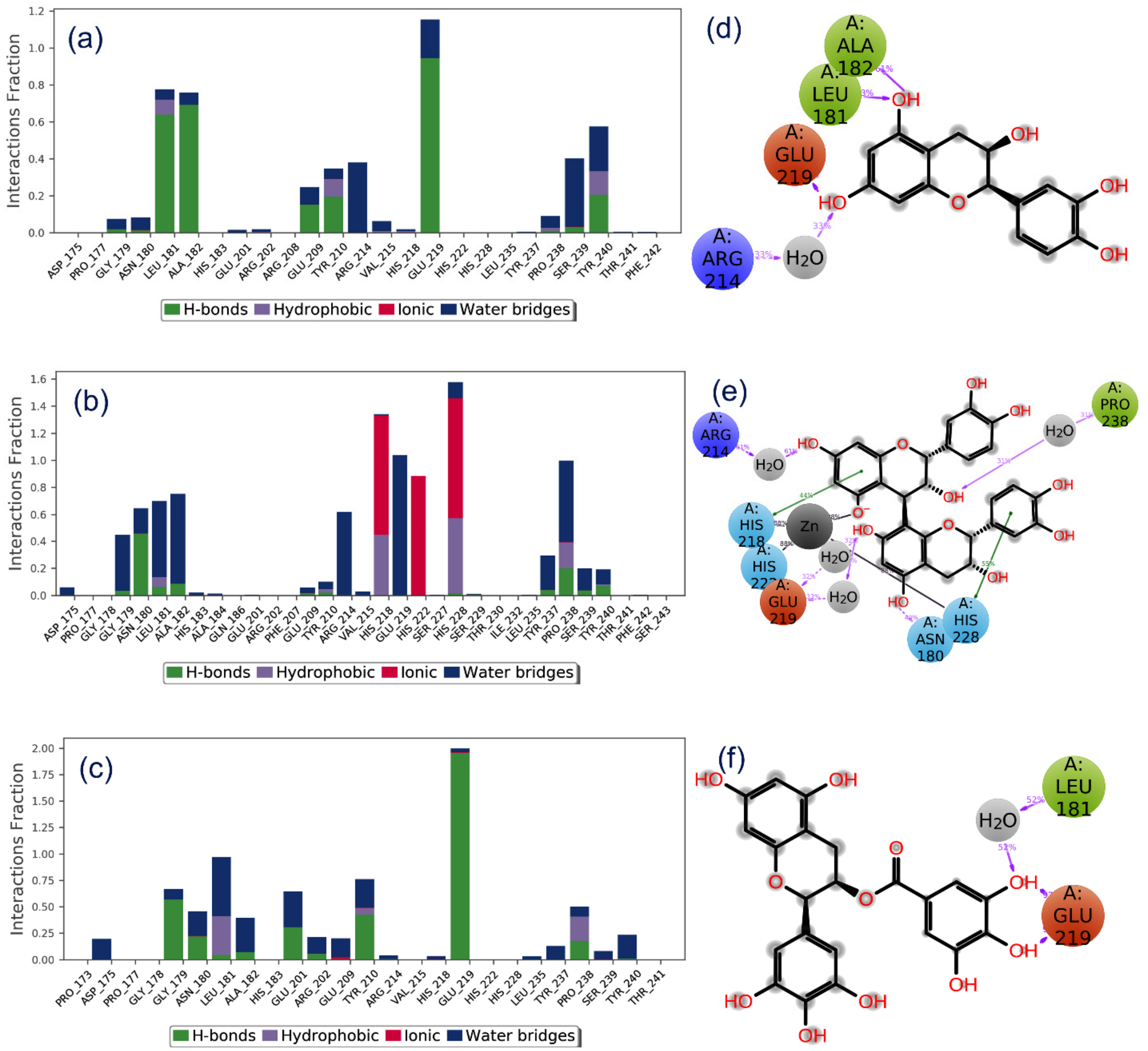

Figure S3. Protein-ligand contact map for (a) MMP-1-(-)-epicatechin, (b) MMP-1-proanthocyanidin B2, and (c) MMP-1-EGCG derived from the respective 500 ns simulation trajectories. Also, schematic representation depicts the interaction between protein and ligand at 30% of the total 500 ns MD simulation interval.

S1.4. Post-molecular simulation quantum chemical calculations

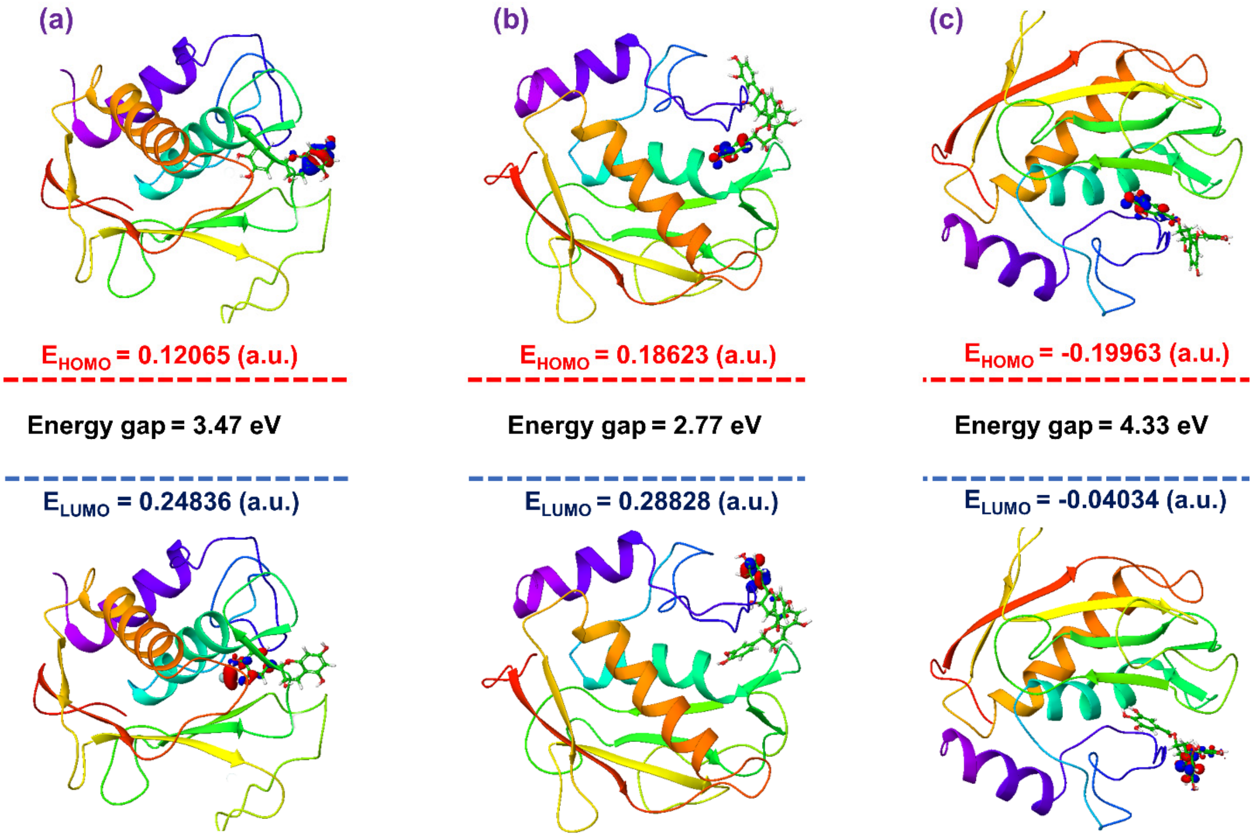

Figure S4. Frontier molecular orbitals, i.e. HOMO and LUMO along with energy values and energy band gap were calculated for the last snapshot from 500 ns MD simulation of molecular docked bioactive compounds (a) (-)-Epicatechin, (b) Proanthocyanidin B2, and (c) reference compound EGCG with MMP-1 using ONIOM(B3LYP/6-31G**:UFF) method.

S1.3. Binding Affinity calculations.

Table S5: Summary of various energy components considered in the free binding energy for the docked and simulated complexes.

Energy components	(-)-Epicatechin		Proanthocyanidin B2		Epigallocatechin Gallate	
	Before MD	After MD	Before MD	After MD	Before MD	After MD
ΔG_{Bind}	-12.19	-28.70±3.82	-26.85	-13.907±5.54	-18.13	-25.53±4.83
$\Delta G_{Bind\ Coulomb}$	-30.24	-28.90±4.2	-43.12	-20.787±9.06	-32.16	-33.8583±7.57
$\Delta G_{Bind\ Covalent}$	6.59	2.02±0.35	6.07	5.949±3.72	10.10	1.85958±1.62
$\Delta G_{Bind\ vdW}$	-15.4	-26.32±1.59	-26.16	-26.229±5.09	-27.26	-30.6873±3.11
$\Delta G_{Bind\ Solv\ SA}$	2.4	0.86±1.28	2.20	7.084±1.11	2.08	0.561±1.48
$\Delta G_{Bind\ Solv\ GB}$	24.5	23.63±2.80	34.16	20.077±6.80	28.23	36.59483±3.54
